# STREAM-PRS: a multi-tool pipeline for streamlining polygenic risk score computation

**DOI:** 10.1186/s13073-025-01539-0

**Published:** 2025-10-09

**Authors:** Sara Becelaere, Yasmina Abakkouy, Deborah Sarah Jans, Margaux David, Séverine Vermeire, Isabelle Cleynen

**Affiliations:** 1 Laboratory for Complex Genetics, Department of Human Genetics, KU Leuven, Leuven, Belgium; 2 Laboratory for Human Evolutionary Genetics, Department of Human Genetics, KU Leuven, Leuven, Belgium; 3 Laboratory for Neuroimmunology, Department of Neurosciences, KU Leuven, Leuven, Belgium; 4 Translational Research in Gastrointestinal Disorders, Department of Chronic Diseases and Metabolism (CHROMETA), KU Leuven, Leuven, Belgium; 5 Department of Gastroenterology and Hepatology, University Hospitals Leuven, Leuven, Belgium

**Keywords:** Polygenic risk scores, Precision medicine, Complex disease, Automated multi-tool pipeline, Inflammatory bowel disease

## Abstract

**Background:**

Polygenic risk scores (PRS) offer an elegant approach to estimating an individual’s genetic predisposition to a given disease or trait. Numerous tools are available for PRS calculation, each applying different strategies to account for linkage disequilibrium and effect size shrinkage. No single tool is inherently superior. Therefore, multiple tools should be tested to identify the one that best suits the research question. Additionally, challenges such as population stratification and PRS portability further complicate the field. Here, we developed STREAM-PRS, a PRS pipeline designed to calculate scores using five popular tools: PRSice-2, PRS-CS, LDpred2, lassosum, and lassosum2.

**Methods:**

STREAM-PRS first computes scores under various settings in a training dataset. The selected variants are subsequently used for score calculation in the test dataset, followed by PC correction and standardization to improve portability across different centers. Finally, the pipeline determines the best PRS tool and settings based on the variance explained (*R*^2^) in the test dataset. To demonstrate this PRS pipeline, we applied it to an in-house inflammatory bowel disease (IBD) cohort consisting of 3192 IBD cases and 822 controls. In total, 472 scores were created using The 1000 Genomes non-Finnish European subpopulation as training data and applied to UK Biobank data as the test dataset.

**Results:**

Using STREAM-PRS for 472 scores across the 5 PRS tools with 404 individuals in the training and 1000 individuals in the test dataset takes approximately 20 h to complete. For IBD, lassosum was identified as the best-performing tool with optimal settings as follows: a shrinkage value of 0.7 and a lambda value of 0.008859. Applying this optimized PRS to our in-house IBD dataset (validation) resulted in an *R*^²^ of 0.203 and an AUC of 0.75. Further, the PRS showed a high positive predictive value of 0.905 but a low negative predictive value of 0.341. This suggests that the PRS is effective in identifying individuals at high risk but might be less reliable in excluding lower risk individuals.

**Conclusions:**

Overall, STREAM-PRS provides an efficient framework for selecting the best PRS calculation strategy and helps bridge the portability gap within the PRS field. STREAM-PRS is available at https://github.com/SaraBecelaere/STREAM-PRS

**Supplementary Information:**

The online version contains supplementary material available at 10.1186/s13073-025-01539-0.

## Background

Genome-wide association studies (GWAS) investigate the relationship between hundreds of thousands of genetic variants (single nucleotide polymorphisms, SNPs) and complex traits or diseases. These studies have been instrumental in identifying SNPs with usually smaller effects that are significantly associated with the trait or disease of interest and collectively contribute to genetic risk. Findings from GWAS help shed light on the biological pathways involved in these complex traits and disorders. For example, a recent GWAS on the complex trait “height” revealed that for populations of European ancestry, the majority of associated variants (over 7000 genome-wide associated loci) tend to cluster nonrandomly in close proximity to one another and to well-known skeletal growth genes [1]. In Alzheimer’s disease (AD), GWAS identified more than 75 susceptibility loci [2], harboring variants associated with several biological pathways affected in AD, such as Aβ and tau processing, cholesterol metabolism, immune response, and endocytosis [3]. Finally, inflammatory bowel disease (IBD), characterized by chronic inflammation of the gastrointestinal tract, consists of two main subtypes: Crohn’s disease (CD) and ulcerative colitis (UC). Over 320 genetic risk loci have been identified through GWAS, with innate immunity, epithelial barrier function, and immune cell activation as some associated biological pathways [4, 5]. Hence, GWAS are a powerful tool to find genetic associations with complex diseases and traits. These findings can then be leveraged for several post-GWAS analyses, such as the calculation of polygenic risk scores (PRS).

In PRS, the effects of associated SNPs from GWAS are combined to estimate an individual’s genetic predisposition to a complex trait or risk of developing a disorder. PRS are relatively straightforward and inexpensive to assess; however, there is no standardized method for calculating them, and several challenges remain. Various software tools are available for calculating PRS, each with their own strengths and weaknesses. These tools primarily differ in how they handle linkage disequilibrium (LD) and how they adjust—or shrink—effect size estimates from GWAS. The traditional approach, clumping and thresholding (C + T), includes only SNPs with GWAS *P*-values below a specific threshold, treating excluded SNPs as having zero effect size while leaving the effect size of included SNPs unchanged (e.g., PRSice-2 [6]). Alternative methods adjust the effect size estimates of all SNPs using statistical techniques such as lasso/ridge regression (e.g., lassosum [7] and lassosum2 [8]) or Bayesian approaches (e.g., LDpred2 [9] and PRS-CS [10].

Most studies that include analyses involving polygenic risk scores typically rely on a single software tool for their calculations. To help researchers identify the most appropriate tool for their analysis, various comparisons between tools have been conducted for different outcomes [11–13]. These comparisons suggest that the traditional C + T approach rarely provides the best results, but on the other hand, no single tool consistently outperforms the others across all outcomes. The optimal method or tool often depends on the Genetic architecture of the trait and the sample size of the discovery GWAS. For example, when comparing 10 different methods for psychiatric disorders in several case-control cohorts, megaPRS, LDpred2, and SBayesR ranked highest in most comparisons and were recommended for psychiatric disorders [12]. In a separate study, Leonenko et al*.* compared six different PRS tools for AD, finding that different methods yielded very similar performance, though individual scores varied across methods [13].

Although guides exist that give pointers on how to calculate PRS [14], using a dedicated PRS pipeline that incorporates multiple state-of-the-art PRS software tools can streamline the process of building and comparing scores. One such pipeline, the polygenic score (PGS) catalog calculator (pgsc_calc), generates multiple PRS by employing different published and available PRS in the PGS catalog for the disease or trait of interest [15]. A distinguishing feature of this tool is its ability to estimate Genetic ancestry and to normalize scores using an external reference dataset, namely The 1000 Genomes Project data [16]. However, its limitation lies in the fact that researchers are restricted to existing PRS; it cannot calculate scores for phenotypes lacking PRS in the PGS catalog.

In this paper, we propose STREAM-PRS (*S*treamlined *T*oolkit for *R*eliable *E*valuation and *A*nalysis of *M*ultiple *P*olygenic *R*isk *S*cores), a PRS pipeline that starts with quality-controlled data to calculate PRS using multiple tools that cover common shrinkage strategies: C + T (PRSice-2), Bayesian shrinkage (PRS-CS, LDpred2), lasso regression (lassosum), and ridge regression (lassosum2). We address challenges such as selecting optimal parameters, determining which SNPs to include, and standardizing the scores. To demonstrate the use of STREAM-PRS, we apply it to the quantitative trait BMI and the binary trait IBD. For the latter, we have an in-house IBD dataset, consisting of patients with CD and UC, and non-IBD controls. With heritability estimates from twin studies of 63% for UC and 75% for CD [17] and over 320 genetic risk loci identified through GWAS [5], IBD serves as an ideal complex genetic disorder for showcasing our PRS pipeline.

## Methods

### PRS pipeline overview

**Fig. 1:**
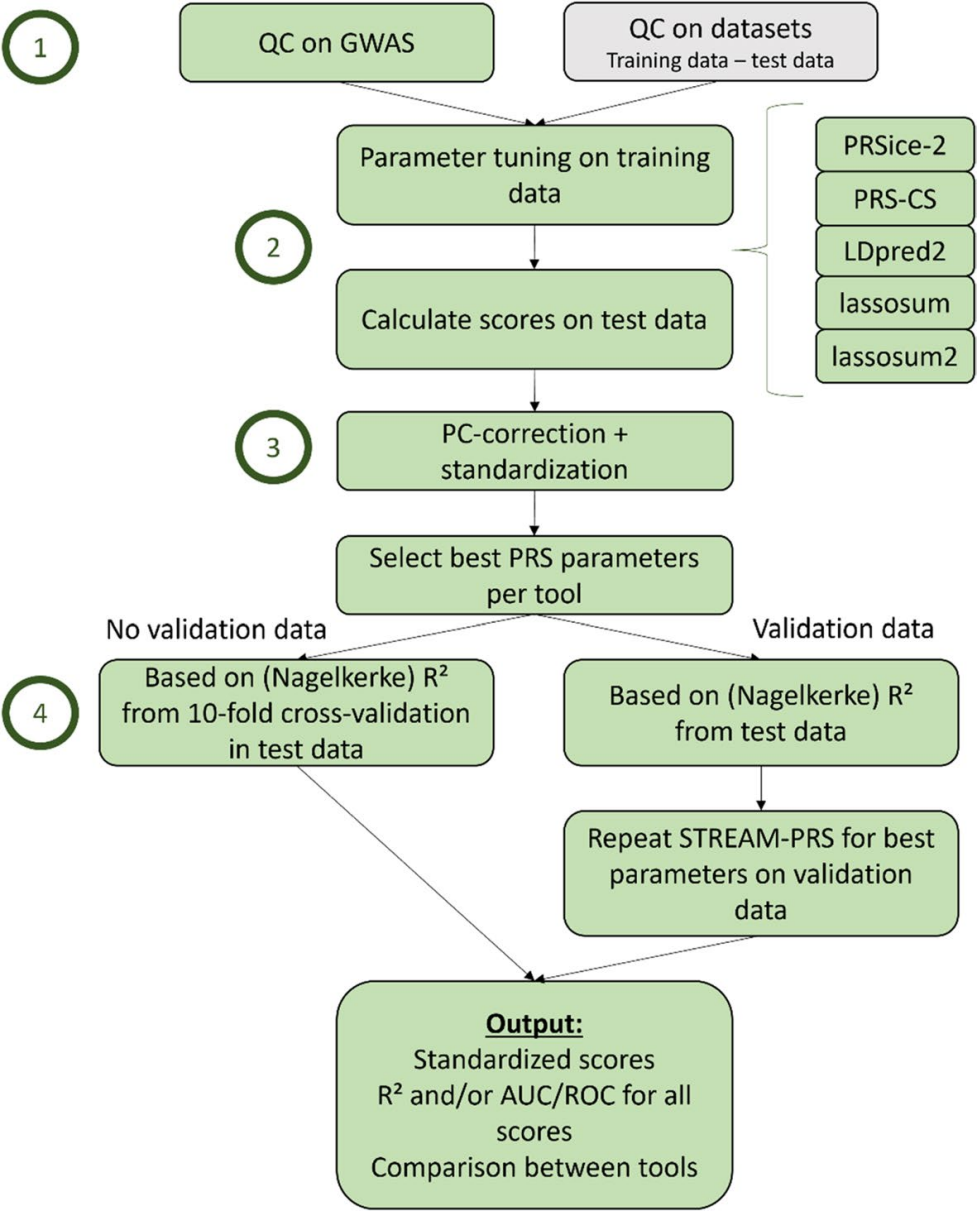
Overview of the STREAM-PRS pipeline. The four main steps of the pipeline are indicated: 1. GWAS QC, which consists of removing ambiguous, multiallelic, and duplicate SNPs, 2. score calculation for the five included PRS tools (PRSice-2, PRS-CS, LDpred2, lassosum, and lassosum2) by tuning the parameters in the training dataset and using these tuned parameters in the test dataset, 3. PC correction and standardization of all scores, and 4. selection of best PRS. In case no validation dataset is available, 10-fold cross-validation is used to obtain the performance metrics. In case a validation dataset is available, STREAM-PRS is repeated for the best parameter settings only, and the performance metrics are obtained from the validation data

STREAM-PRS [18] is composed of four steps (Fig. 1). The first step consists of quality control (QC) of the GWAS summary statistics (base file). In the second step, scores are calculated using different software tools: PRSice-2, PRS-CS, LDpred-2, lassosum, and lassosum2 [6–10]. These tools were chosen because they are commonly used and employ different methods for score calculation, including clumping + thresholding (C + T), Bayesian methods, and lasso/ridge regression. A range of parameter settings can be chosen for each tool, and in a later step of the pipeline, the best settings are determined per tool. Importantly, the scores are first built on a training dataset to tune the hyperparameters and then applied to a test dataset. In the third step, all scores undergo principal component correction (PC correction) and standardization based on the training dataset. Finally, in the fourth step, the best parameters are selected per tool, and the overall best PRS is chosen by comparing variance explained (*R*^2^) and area under the receiver operating characteristic (ROC) curve (AUC). Optionally, these best parameters can be used to rerun the pipeline on a validation dataset to obtain the final *R*^2^ and AUC for the PRS of the trait or disease of interest. In case no validation dataset is available, 10-fold cross-validation can be used. Details of each step are given below.

## Data preparation—quality control GWAS

The GWAS summary statistics file undergoes QC, which involves removing ambiguous SNPs (C/G and A/T SNPs), multiallelic SNPs, and duplicate SNPs. Furthermore, the pipeline ensures correct formatting of numerical values, such as *P*-values. As part of this step, STREAM-PRS generates three new GWAS files, each formatted specifically for the five PRS calculation tools: one file for PRSice-2 and lassosum, one for PRS-CS, and one for LDpred2 and lassosum2. Note that the pipeline does not perform QC on the training, test, and validation datasets. Users are responsible for applying basic QC steps to these datasets, such as filtering for minor allele frequency (MAF) and missingness [19], which allows them the freedom of adapting these QC steps according to their own insights and research purposes.

## PRS calculation and parameter settings

After QC and formatting of the GWAS, PRS are calculated using the five incorporated tools across various parameter settings [6–10]. STREAM-PRS was designed to provide users with flexibility in choosing their parameter settings for PRS calculation. It can be used to calculate scores for a single parameter when the optimal settings for the trait of interest are already known or to explore a wide range of parameter combinations to find the best settings for each tool. The adjustable parameters—determined by the functionalities provided by each tool—include *P*-value thresholds (Pts) and clumping parameters (for PRSice-2), shrinking values (for PRS-CS, lassosum, lassosum2), LD block reference files (for PRS-CS, lassosum), and a range of heritability estimates (h2) and proportion of causal variants (p) (for LDpred2). Table 1 provides the specific parameters that can be customized for each tool, along with the pipeline default parameter settings used in our use cases with the BMI and IBD PRS. In total, we calculated 8 scores for PRSice-2, 4 for PRS-CS, 200 for LDpred2 (infinitesimal, grid, auto grid, and auto model are used), 140 for lassosum, and 120 for lassosum2, resulting in 472 scores in total (Table 1).

**Table 1:** Customizable parameters for each tool along with parameter settings used for our use cases (BMI and IBD PRS)

Software tool	Parameter	Chosen settings for use cases (default parameters)
**PRSice-2** Eight scores	*P*-value threshold (Pt)	5e-08, 1e-05, 0.0001, 0.001, 0.05, 0.1, 0.5, 1
	Clumping	Blocks = 250 kb*, *r*^2^ = 0.1*, *P*-value = 1*
**PRS-CS** Four scores	Phi-values	1e + 00*, 1e-02*, 1e-04*,1e-06*
	LD block reference	ldblk_ukbb_eur
**LDpred2**—inf model One score	/	/
**LDpred2**—grid model One-hundred sixty-eight scores	Heritability estimate (h2)	Heritability (estimated with snp_ldsc2 function) multiplied by 0.3*, 0.7*, 1*, or 1.4*
	Proportion of causal variants (p)	R expression: signif(seq_log(1e-5, 1, length.out = 21), 2)
	Sparse model used	TRUE or FALSE*
**LDpred**—auto grid model Thirty scores	Initial values for *p*	R expression: seq_log(1e-4, 0.2, length.out = 30)*
**LDpred2**—auto model One score	Starts from LDpred2 auto grid model parameters	/
**Lassosum** One-hundred forty scores	Shrinkage	0.1, 0.2*, 0.4, 0.5*, 0.7, 0.9*, 1*
	Lambda values	R expression: exp(seq(log(0.001), log(0.1), length.out = 20))*
	LD block reference	EUR.hg38
**Lassosum2** One-hundred twenty scores	Delta	0.001*, 0.01*, 0.1*, 1*
	Lambda values	Thirty different lambda’s, with a ratio of 0.01 between the last and first lambda to try*

*These parameters are recommended by the authors of the corresponding tool [6–10]

## PC correction and standardization

After calculating scores across all parameter settings and tools, the STREAM-PRS pipeline identifies the optimal parameter settings for each tool (see the “Statistical methods: selection of the best scores” below). These best scores are then compared across different tools to determine the overall best score. Prior to this comparison, the scores undergo PC correction to adjust for population substructure and standardization, where we use a similar method as in Khera et al*.* [20]. PC correction involves regressing out a predefined number (customizable by the user) of PCs from the scores. Following PC correction, the scores are standardized relative to the scores of the training dataset by subtracting the mean score of the training dataset from each individual’s score in the test dataset and then dividing this value by the standard deviation (SD) of the scores in the training dataset.

## Statistical methods: selection of the best scores

To identify the best scores for each tool, logistic or linear regression analyses are conducted for all 472 calculated scores. The (pseudo-)*R*^2^ value, specifically Nagelkerke’s *R*^²^ and Cox-Snell *R*^2^ for binary traits and adjusted *R*^*2*^ for quantitative traits, is determined. The score corresponding to the highest Nagelkerke/adjusted *R*^*2*^ is selected as the best score, provided that the regression model is statistically significant (*P*-value < 0.05). For the selection of the overall best score in the case of a binary outcome trait, besides the pseudo-*R*^²^ value, the area under the receiver operating curve (AUC) can also be taken into account. When no validation dataset is available, we recommend users to use the 10-fold cross-validation option in the pipeline. In this case, the best scores are determined by the highest *R*^²^ value for quantitative traits and by the highest AUC for binary traits as implemented in the R “caret” package (v.6.0–94).

## Generated output

The pipeline generates various output files. First, it produces text files that contain raw and standardized scores (i.e., PC corrected and standardized) for all individuals, which were computed by each tool across all predefined parameter settings. In addition, STREAM-PRS creates files containing only the best score for each tool for all individuals. Regression result files are also produced, including essential statistical metrics such as beta coefficients, standard error (SE), odds ratio (OR), 95% confidence intervals (CI), *P*-values, and *R*^²^ values derived from the regression models. The regression results for the best parameter settings for each tool are combined into a single, separate file. The pipeline also reports AUC values for the scores with the best parameter settings for each tool. Finally, to aid visualization and interpretation, the pipeline generates several types of plots. These include histograms, density plots, and boxplots to display the distribution of the best PRS per tool across cases and controls, barplots showing *R*^²^ values for each tool and across tools, and a ROC curve plot for the best PRS per tool. All statistical analyses and visualizations are performed in R v4.2.2, using the ggplot2 v3.4.2, dplyr v1.1.2, DescTools v0.99.50, caret v.6.0–94, and pROC v1.18.5 packages.

## Implementation details

The five tools used in the pipeline were developed in different programming languages (R and Python). To streamline their execution, we created a unified bash script, STREAM-PRS.bash, which sequentially runs the tools. This main bash script executes seven different scripts: edit_GWAS.r, prsice.bash, lasso.r, PRScs.bash, LDpred2_and_lassosum2.r, and get_best_PRS.r. The last script, get_best_PRS.r, has two versions—one for binary traits and one for quantitative traits. Users specify in STREAM-PRS.bash whether their trait is binary or quantitative. All other user-definable parameter settings are directly adaptable in the STREAM-PRS.bash script.

To run the full pipeline, we made use of the Flemish Supercomputer infrastructure and used one IceLake node, which consists of 2 Intel Xeon Platinum 8360Y CPUs at 2.4 GHz (IceLake) with 36 cores each and has 256 GiB RAM.

### Additional analyses

For the IBD use case, we further validated the best-performing score using a separate validation set. For this purpose, we included logistic regression and ROC analyses, following the methods described previously. Statistical differences between AUC values were calculated with the DeLong test (pROC package), and *P*-values were adjusted for multiple testing (Bonferroni). Other additional analyses that we performed include the following: 1. Determining the correlation between the different best scores, 2. ranking individuals based on their PRS values and evaluating the differences between tools, and 3. calculating prediction metrics derived from a confusion matrix, such as sensitivity, specificity, positive predictive value, and negative predictive value. Statistical significance of the correlations was tested with the cor.test function. To determine whether the overlaps in scores were non-random, we used Fisher’s exact test. *P*-values from both statistical tests were adjusted for multiple testing with Bonferroni correction. A *P*-value < 0.05 was considered statistically significant. All analyses were done using R v4.2.2.

### Cohorts and input data for the use cases

Below, we give details about the input data that was used to demonstrate STREAM-PRS. We used the pipeline on two use cases: the continuous trait BMI and the binary trait IBD. Since BMI information was only available in the test dataset (UK Biobank) and not in our in-house validation dataset, we performed 10-fold cross-validation. For IBD, an in-house validation dataset was available. An overview of the datasets and the specific workflow that was followed for the use cases is given in Additional file 1: Fig. S1.

## Base GWAS

For the BMI PRS computation, summary statistics from Locke et al*.* (2015) were used [21], which is the most recent BMI GWAS that does not include UK Biobank individuals. Ambiguous SNPs—A/T and G/C variants—were removed, and additional filtering and formatting steps were incorporated into the PRS pipeline, as outlined above. The summary statistics from de Lange et al*.* were used as the base data for the IBD PRS calculations [22]. While this GWAS is not the most recent or largest for IBD, it was chosen as it is independent of our dataset and primarily includes individuals of European ancestry. The same filtering was applied as for the BMI GWAS, and Genome positions were converted from Genome build 37 to 38 with LiftOver [23]. Some of the tools in the pipeline required extra information not originally present in the summary statistics. For instance, lassosum needed a column indicating the sample size per variant (effective sample size), while PRS-CS and LDpred2 required rsIDs. Therefore, additional columns with the number of individuals in the original GWAS (*N* = 59,957) and rsIDs were added to the summary statistics. RsIDs were derived from dbSNP v155 [24]. When effective sample size and rsIDs are present in the summary statistics, we recommend using these columns instead, as was done for the BMI GWAS.

## Training data—1000 Genomes

The pipeline uses a training dataset in PLINK [25] format (.bed,.bim,.fam) to determine which SNPs are included in the scores. For PRSice-2, this dataset also served as the reference for clumping. We chose the non-Finnish European subset from The 1000 Genomes Project (1 KG-NFE, *N* = 404) as our training dataset [16]. The primary reason for this choice is the population similarity with our test dataset (UK Biobank), our validation dataset (a Belgian cohort), and the base GWAS used, which predominantly includes individuals of European ancestry. Only SNPs from autosomal chromosomes and *MAF* > 0.01 were included. Since the training dataset is used for parameter tuning, only SNPs that were selected from this dataset are used for the PRS calculation in the test dataset. Therefore, to ensure sufficient overlap between datasets, only SNPs present in HapMap3 were included [26]. However, this restriction to HapMap3 SNPs is optional and is not hardcoded in the pipeline.

## Test data—UK Biobank

As the test dataset, PLINK files in.bed,.bim, and.fam format were used as input files. For this study, we used the imputed genotype data from the UK Biobank (release November 2022) [27]. Genome positions in the imputed dataset were converted from Genomic build 37 to build 38 using LiftOver [23]. Variants with a *MAF* < 0.01 and an INFO score < 0.3 were removed. Individuals were excluded if there was a mismatch between reported and genetic sex, if they were outliers for missingness or heterozygosity, or if they did not self-identify as white British [27]. Individuals were also filtered for relatedness. Relatedness was assessed using IBIS [28] across the entire UK Biobank dataset, using a minimum segment length of 10 cm and the --maxDist parameter. For this analysis, the unimputed dataset containing 713,747 SNPs was used. One individual from each pair of related participants was removed if IBIS identified them as either first- or second-degree relatives. Furthermore, variants and individuals with more than 5% missingness were excluded. This resulted in 357,622 individuals and 7,328,047 variants left for further analyses.

For the IBD analysis, individuals were identified as having IBD if they had one of the ICD-10 codes for Crohn’s disease (K500, K501, K508, K509) or ulcerative colitis (K510, K511, K512, K513, K514, K515, K518, K519). Individuals with other noninfective gastroenteritis and colitis (K520, K521, K522, K523, K528, K529) were excluded. With these criteria, there were 4126 cases in the UK Biobank dataset that passed the QC as described above.

## Validation data—IBD dataset

The validation dataset used in the IBD application is an in-house IBD dataset comprising a sporadic case-control cohort recruited through the IBD unit of the University Hospital Leuven (Belgium) as part of the IBD Genetics study. This dataset included 3192 IBD cases and 822 controls, which are independent from the base GWAS, training data, and test data.

Individuals in this dataset were genotyped using the Infinium Global Screening Array-24 Kit (Illumina) with added customizable content, totaling up to 688,032 SNPs. Quality control was performed as per IIBDGC workflow [29]. First, indels, monomorphic variants, and mitochondrial variants were removed. Samples were excluded if they either1. had a call rate < 95%, 2. had a heterozygosity rate exceeding four standard deviations, 3. had non-European ancestry, or 4.were duplicates. SNPs were removed if they 1. had a call rate < 98%, 2. had a *MAF* < 0.01, 3. showed a deviation from Hardy–Weinberg equilibrium in controls with *P*-value < 1e-5 4. or with *P*-value < 1e-12 in cases, 5.were ambiguous with a MAF > 0.45, or 6. were not present in TOPMed. Imputation was performed with TOPMed as the reference. After imputation, SNPs were excluded if they 1. had an *R*^2^ value < 0.4, 2. had *MAF* < 0.01, 3. had a Hardy–Weinberg equilibrium in controls with *P*-value < 1e-5, 4. showed a deviation from Hardy–Weinberg equilibrium in cases with *P*-value < 1e-12, or 5. were located on the X chromosome. After QC, the validation dataset consisted of 3840 individuals (3042 cases and 798 controls) and 8,772,092 SNPs.

## Other input files

Finally, STREAM-PRS also requires three other files as input: 1. A PC file for the training dataset, 2. a PC file for the test dataset, and 3. a phenotype file. The PC files should contain the FID and IID of the individuals and their corresponding PC values, which are used for the PC-correction step (see above). For both our use cases, we used the first five PCs that were computed by projecting the data onto the 1 KG-NFE subset of The 1000 Genomes using PLINK and PLINK2 [25, 30]. The phenotype file only contains FID, IID, and the phenotype of the individuals in the test/validation dataset.

### GWAS and LD score regression for IBD

To assess whether IBD exhibits measurable heritability in both the UK Biobank and our in-house dataset, we conducted genome-wide association studies (GWAS) for IBD in each cohort using SAIGE with default parameters [31]. Subsequently, we estimated SNP-based heritability using LD score regression (LDSC) [32]. For this analysis, we restricted to HapMap3 SNPs and excluded the extended MHC region due to its complex LD structure.

## Results

### STREAM-PRS performance and parameter selection

Below, we go into detail on how we incorporated the different tools into the pipeline. We specifically address the challenges encountered during this process, the criteria and methods used to select the SNPs included in the scores, and the approach taken to standardize the calculated scores for consistency and comparability.

#### Addressing computational challenges

During the development of STREAM-PRS, we encountered several technical hurdles that required optimization. One significant challenge was the inclusion of five tools developed in different programming languages (R and Python), which we integrated in a unified bash script that sequentially runs the tools. While this ensured smooth execution, it introduced a trade-off by increasing the total runtime compared to running the tools simultaneously (~ 25 h vs. ~ 20 h for 1000 samples, Additional file 2: Table S1). However, this runtime increase is moderate, considering that most tools need less than 1 h to run, while PRS-CS takes ~ 20 h regardless (Additional file 2: Table S1). Attempts to run the tools concurrently often resulted in the system running out of memory (Additional file 2: Table S1), primarily due to PRS-CS, which has high memory and computational demands. Given these resource-intensive requirements, we recommend running STREAM-PRS on a full compute node (consisting of 72 cores in our case) with sufficient memory and processing power (see the “Methods” for our setup).

#### Formatting and quality control of GWAS summary statistics

The editing and formatting of GWAS summary statistics in STREAM-PRS were tailored to meet the different requirements of the different PRS tools. Each tool imposes specific constraints on the structure and content of input files. For example, PRS-CS requires summary statistics with columns SNP, A1, A2, BETA/OR, and SE and a header with these exact labels. Conversely, PRSice-2 does not require a specific header but mandates the inclusion of *P*-values in the summary statistics file. To address these differences, we developed a preprocessing script that standardizes and formats the GWAS summary statistics to ensure compatibility with each tool. This script generates three separate files: one for PRSice-2 and lassosum, one for PRS-CS, and one for LDpred2 and lassosum2. In addition to the formatting, this script also performs some quality control steps (see the “Methods”).

Some tools presented additional challenges related to genome alignment and variant annotation. While certain tools (PRS-CS, LDpred2, and lassosum2) require rsIDs or accept chromosome position coordinates only in Genome build 37 for input, others (PRSice-2 and lassosum) accept chromosome position coordinates in build 38 as well. Our base dataset was aligned to Genome build 38 and lacked rsIDs, necessitating conversion to rsID format. However, since not all variants had corresponding rsIDs, we retained a version annotated with chromosome positions to maximize the number of variants included. As a direct result of these considerations, the pipeline requires two versions of all input files (GWAS summary statistics, training data, testing data): one annotated by rsIDs and one annotated by chromosome position. This strategy ensured compatibility with all tools while preserving as much data as possible for downstream analyses.

#### Inclusion of HapMap3 SNPs to enhance reproducibility

Another key consideration was to decide which variants to include in the scores. There are many correlated SNPs present in GWAS summary statistics, and the five PRS tools each handle LD in different ways. PRSice-2 employs C + T, lassosum and lassosum2 use penalization, and PRS-CS and LDpred2 apply Bayesian methods to shrink the effect sizes of correlated SNPs. To improve the reproducibility of the calculated PRS, we opted to tune the hyperparameters on a training dataset (here, 1 KG-NFE). This approach is more objective, as it relies on the LD structure of a reference population rather than a potentially biased case/control dataset. In addition, PRS-CS, LDpred2, and lassosum2 inherently use only SNPs from the HapMap3 dataset [26] for their shrinkage and penalization steps, as predefined LD blocks or LD correlation matrices are based on this SNP set. Recognizing the importance of standardization for clinical applications, we investigated the impact of limiting all tools to HapMap3 SNPs. This ensures a maximized and consistent set of SNPs across the training (1 KG-NFE), test (UK Biobank), and validation (IBD) datasets.

The overlap with HapMap3 SNPs proved especially beneficial for tools like PRSice-2 and lassosum, which do not inherently restrict to HapMap3 SNPs. Without overlapping with HapMap3 SNPs, many SNPs selected from the training dataset were absent in the validation dataset (Table 2). This was especially pronounced at higher *P*-value thresholds, where the difference in overlapping SNPs between the training and the validation datasets impacted the performance of the PRS in terms of *R*^²^. For example, at a Pt of 1, only 77.84% of SNPs overlapped without applying the HapMap3 filter, resulting in an *R*^²^ of 0.075. However, with HapMap3 overlap, the overlap increased to 97.52% of SNPs overlapped, improving the *R*^²^ to 0.106. Importantly, this step is not built into the PRS pipeline itself, allowing users the flexibility to choose whether to apply this approach or use alternative SNP selection strategies based on their research needs.

**Table 2:** Number of included SNPs for each Pt in PRSice-2 with or without overlap HapMap3 overlap

	**No overlap with HapMap3**			**Overlap with HapMap3**		
**Pt in PRSice-2**	**SNPs in 1 KG-NFE**	**SNPs in IBD dataset (overlap with 1 KG-NFE (%))**	***R***^**2**^	**SNPs in 1 KG-NFE**	**SNPs in IBD dataset (overlap with 1 KG-NFE (%))**	***R***^**2**^
5.00e-08	282	264 (93.62)	0.130	199	197 (98.99)	0.119
1.00e-05	689	652 (94.63)	0.145	454	450 (99.12)	0.133
1.00e-04	1470	1379 (93.81)	0.151	842	836 (99.29)	0.144
1.00e-03	4479	4086 (91.23)	0.139	2033	2026 (99.66)	0.149
5.00e-02	53,700	45,508 (84.74)	0.086	20,391	20,122 (98.68)	0.123
1.00e-01	85,134	71,065 (83.47)	0.082	32,021	31,540 (98.50)	0.112
5.00e-01	227,780	182,388 (80.07)	0.076	86,309	84,477 (97.88)	0.106
1.00e + 00	309,189	240,673 (77.84)	0.075	117,967	115,036 (97.52)	0.106

#### PC correction and standardization

After calculating the scores for the five different tools, we addressed the challenge of finer scale population stratification and score standardization. We wanted the scores to not only be comparable across tools and datasets (standardization) but also to account for differences arising from subtle population differences. We therefore implemented a PC correction and score standardization method based on Khera et al*.* [20] (see the “Methods” for details).

**Fig. 2:**
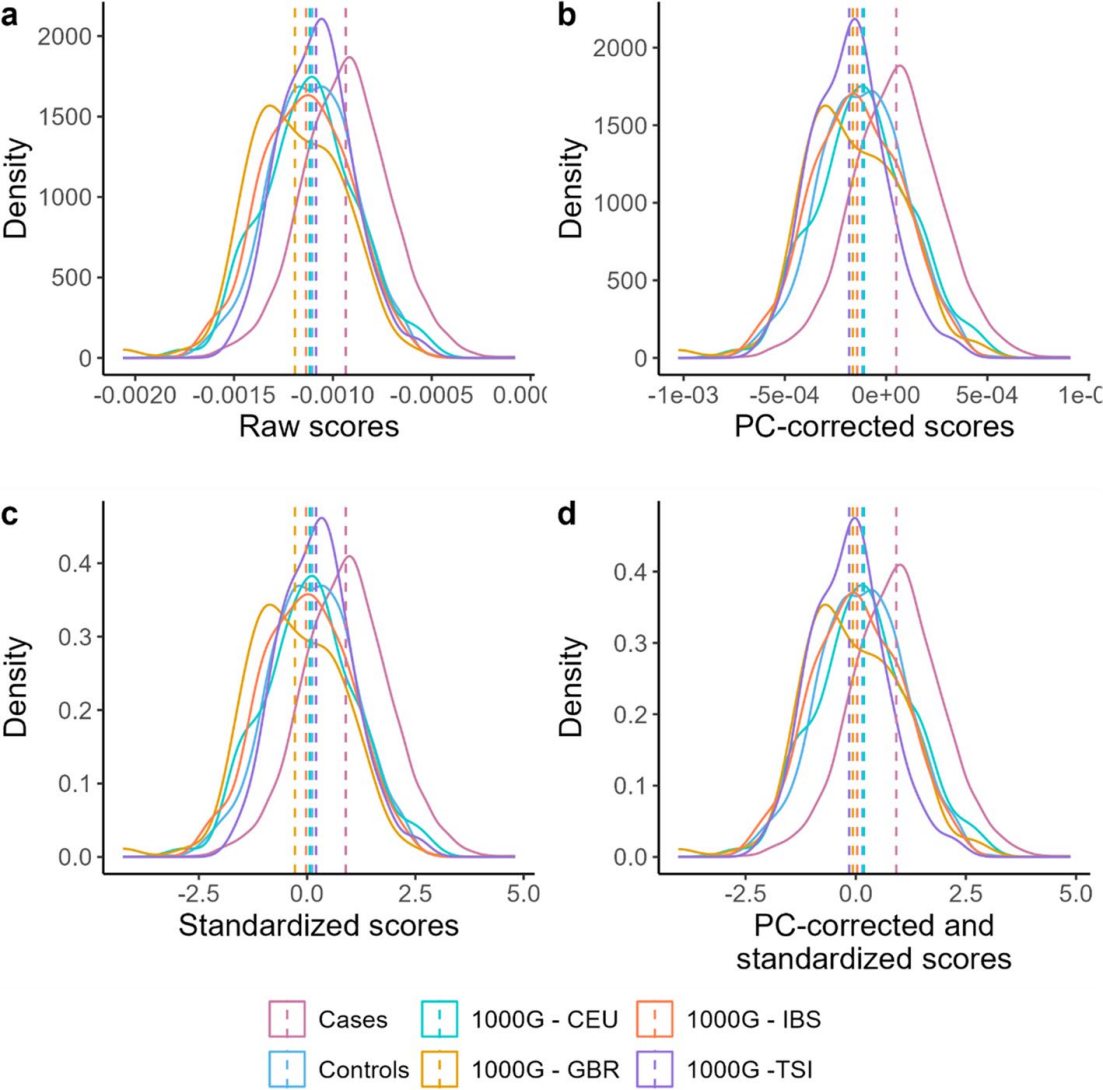
Density curves of the individual scores of the 1 KG-NFE and the IBD dataset. **a** Raw scores, **b** PC-corrected scores, **c** standardized scores, **d** PC-corrected and standardized scores. The scores shown were calculated with PRSice-2. CEU, Northern Europeans from Utah; GBR, British in England and Scotland; IBS, Iberian populations in Spain; TSI, Tuscan in Italy. Cases, individuals with IBD from in-house dataset. Controls, individuals without IBD from in-house dataset

Figure 2 shows the distribution of the scores before and after PC correction, as well as with and without standardization. When examining the score distribution within the 1 KG-NFE population, we observed that while the standardized scores were centered around zero, subpopulations within 1 KG-NFE did not (approximately) share the same mean when no PC correction was performed (Fig. 2c). Especially, the mean score for individuals from the GBR population showed a marked deviation from the other scores, demonstrating that standardization alone does not fully address population stratification. This highlights the need of appropriately addressing genetic ancestry in PRS calculations, even when the target population consists solely of individuals of European ancestry. By regressing out the first five PCs, the scores were adjusted for finer-scale population substructure, i.e., the mean scores of the different subpopulations approximately shared the same mean (Fig. 2b). Subsequent standardization ensured that the scores could be compared across different datasets and tools by bringing the mean scores to zero with a standard deviation of one (Fig. 2 d).

### Use case: application of STREAM-PRS to BMI

To demonstrate the application of STREAM-PRS to a continuous trait, the pipeline was used to calculate scores for BMI, using the parameter settings given in Table 1. In total, 8 scores were generated for PRSice-2, 4 scores for PRS-CS, 200 scores for LDpred2, 140 scores for lassosum, and 120 scores for lassosum2, so 472 scores in total. These scores were subsequently applied to the test dataset, UK Biobank, and the best parameter settings for each tool and overall were selected based on the variance explained (*R*^2^). Since no validation dataset was available for BMI, we applied 10-fold cross-validation (Additional file 1: Fig. S1a). The best-performing tool for BMI was lassosum, which achieved an adjusted *R*^2^ of 0.086 (*P*-value < 3.4e-314). We also built ROC models where we predicted whether individuals developed obesity (BMI > 30) or not. This resulted in an AUC of 0.65 (*CI*: 0.65–0.66). Finally, there was a correlation of 0.292 between the best PRS and the phenotypic BMI (*P*-value < 2.2e-16). All results are presented in Additional file 2: Table S2.

When we correlate the best score obtained from each different PRS tool integrated in STREAM-PRS, the highest correlation was observed between PRS-CS and lassosum (92.4%), which are the two best-performing tools. We also investigated the overlap of individuals in the top and bottom 5% PRS ranges and observed limited overlap between the different tools, Generally below 50%. Of the top 5% scoring individuals identified by lassosum, 81.8% were also in the top 5% of at least one other tool, and 6.6% were identified in the top 5% of all tools (Additional file 2: Table S3).

### Use case: application of STREAM-PRS to IBD dataset

**Fig. 3:**
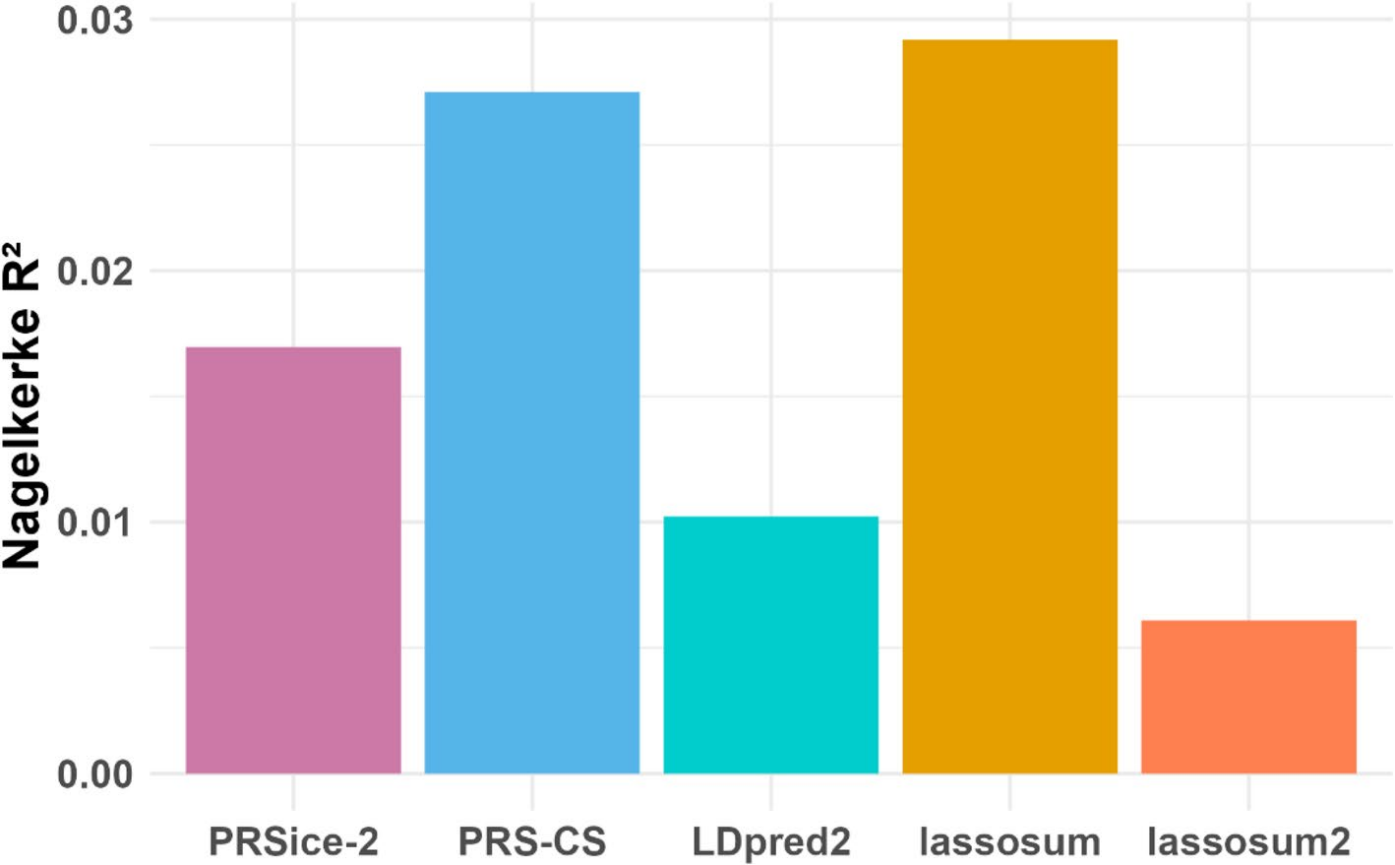
Comparison of *R*^2^ per tool on the test dataset (UK Biobank)

For the IBD use case, again 472 scores were built using the tools and parameters as described above. The best parameter settings for each tool and overall were selected based on the variance explained (Nagelkerke *R*^2^) (Additional file 1: Fig. S1b). The *R*^²^ values showed some variation within individual tools, but when taking into account the scale of the values, this variation is rather small (ranges of *R*^2^ for PRSice-2 (0.011–0.017), PRS-CS (0.015–0.027), LDpred2 (0.0002–0.010), lassosum (0.001–0.029), and lassosum2 (0.001–0.006) (Additional file 2: Table S4). Figure 3 shows the comparison of the *R*^²^ values for each tool, where only the best parameter settings for each are shown. Among the tools, lassosum performed the best, explaining approximately 3% of the variance in IBD, with the PRS being higher in IBD cases than in controls (*R*^2^ = 0.029; *OR* = 2.09 (*CI*: 2.01–2.18); *P*-value = 5.85e-270). The optimal settings for lassosum were a shrinkage value of 0.7 and a lambda value of 0.008859, which resulted in 39,338 SNPs from the 1 KG-NFE data having nonzero shrunk effect sizes. Of these, 31,914 SNPs were present in the UK Biobank dataset.

To evaluate whether the relatively low *R*^²^ values observed in our analysis were driven by the low case-to-control ratio in our dataset (approximately 1:100), we increased the case-to-control ratio to 1:2 by randomly selecting 10 different subsets of control individuals and repeated the logistic regression analysis. These analyses consistently identified the same parameter settings as optimal for lassosum, regardless of the subset of controls that was used (Additional file 1: Fig. S2, Additional file 2: Table S5). Furthermore, the optimal parameters matched those determined using the full UK Biobank dataset. The maximum *R*^²^ achieved in the subset analyses was 0.090, compared to 0.029 when the full dataset was used, suggesting that the low *R*^²^ is primarily driven by the low case-to-control ratio. Similar patterns were observed for the other tools (Additional file 1: Figs. S3, S4, S5, S6, Additional file 2: Tables S6, S7, S8, S9).

**Fig. 4:**
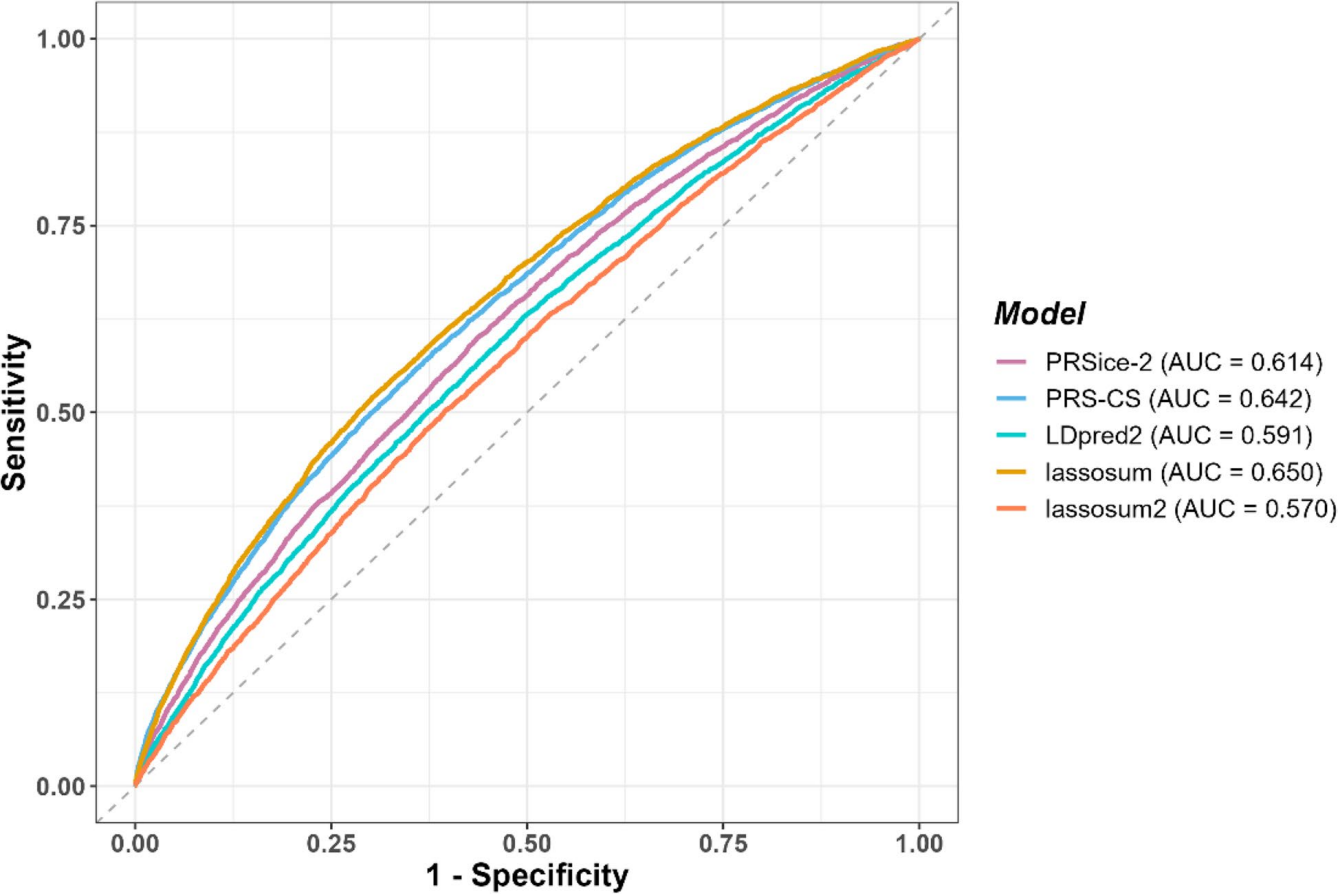
ROC curve of UK Biobank data, predicting IBD cases vs. controls

For the prediction of IBD status, it was also lassosum that performed best in distinguishing between IBD cases and controls, achieving an AUC of 0.65 (*CI*: 0.64–0.66) (Fig. 4). The downsampling analyses were similarly performed for all models, comparing AUC values across different control subsets (Additional file 2: Tables S5, S6, S7, S8, S9). These analyses confirmed that the same parameters were consistently identified as optimal, regardless of the subset used, and both *R*^²^ and AUC identified identical parameter settings as optimal. We also tested a different pseudo-*R*^²^ measure, namely Cox-Snell *R*^²^, which is less dependent on case-to-control ratio (Additional file 2: Tables S4, S5, S6, S7, S8, S9). With this pseudo-*R*^2^, the same parameters were identified as optimal in the UK Biobank data (test data) across all subsets. These values are lower, as expected, but led to the same conclusions.

**Fig. 5:**
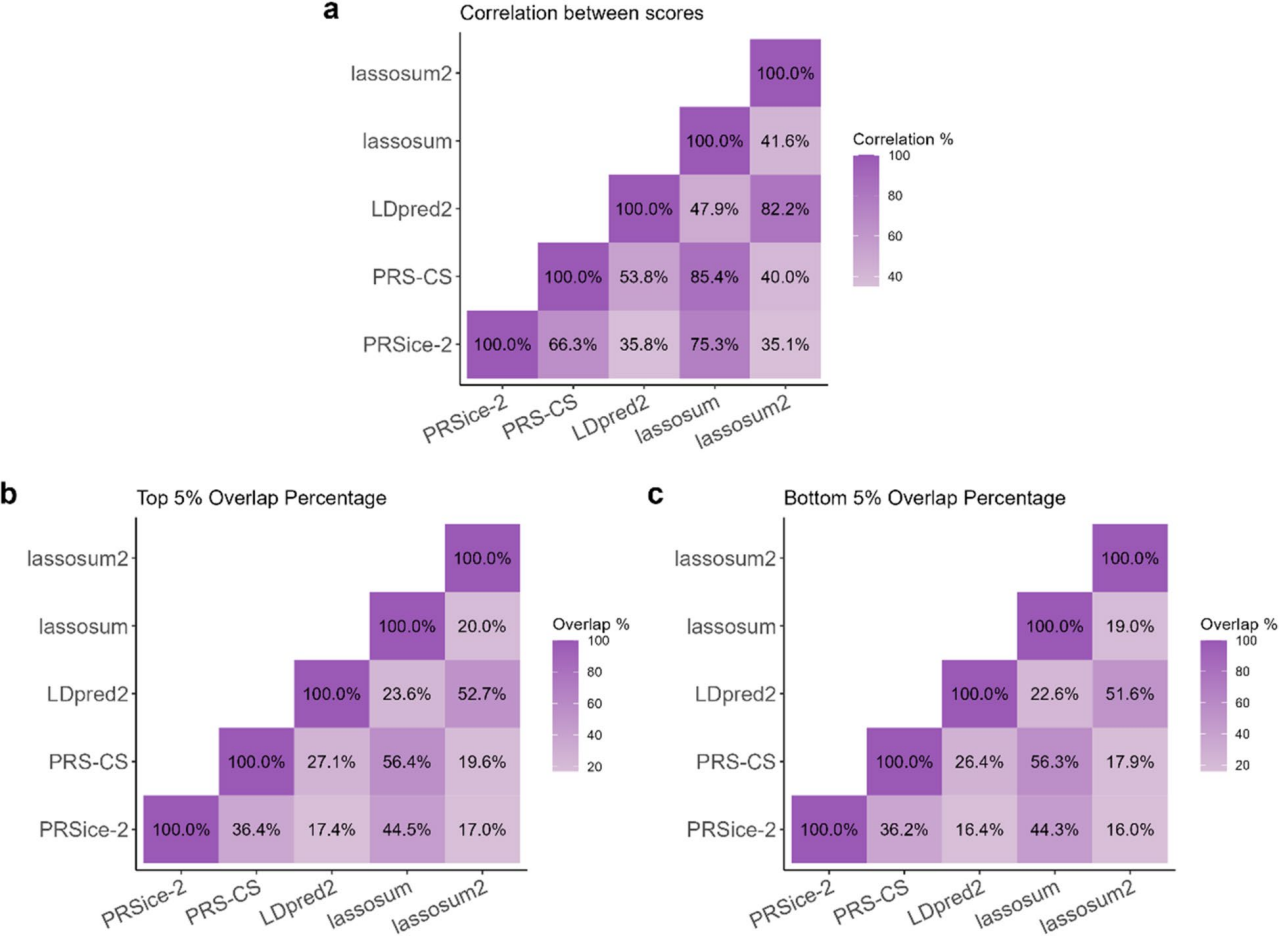
Heatmaps displaying pairwise comparisons of the five tools. **(a)** Correlation between all scores. **(b)** Overlap between the top five percent scoring individuals. **(c)** Overlap between the bottom five percent scoring individuals

Lassosum and PRS-CS had similar performance in predicting IBD status, achieving AUC values of 0.650 and 0.642, respectively. Despite their closeness, the difference was statistically significant (Bonferroni corrected *P*-value = 3.25e-03) (Fig. 4). Notably, the scores derived from these two tools also exhibited the highest correlation with each other (85.4%, Fig. 5a). A similarly high correlation (82.2%) was observed between LDpred2 and lassosum2 scores, which is expected as both tools rely on the same SNP correlation matrix for their computations. In contrast, most other pairwise correlations between scores derived from different tools were relatively low, Generally below 70% (*P*-value < 2.2e-16 for all correlations).

Further analysis of individual rankings across PRS revealed limited overlap between the top and bottom 5% of individuals ranked by their scores across the five tools (Fig. 5b, c, Additional file 1: Fig. S7). Similarly, as for the correlations between the scores, the highest overlaps were observed between the two best-performing tools (lassosum and PRS-CS), as well as between the two least predictive tools (LDpred2 and lassosum2). Specifically, lassosum and PRS-CS shared 56.4% of individuals in the top 5% and 56.3% in the bottom 5%, while LDpred2 and lassosum2 shared 52.7% and 51.6% of individuals in the top and bottom 5%, respectively (*P*-value < 2.2e-16 for all overlaps).

We also examined how many of the top-scoring individuals were IBD cases (Additional file 2: Table S10, Additional file 1: Fig. S8). Of The 4126 cases, 584 were in the top 5% lassosum scores. In other words, 14% of the cases were present in the high-risk group. Among these cases in the top 5% lassosum scores, 83.9% also ranked in the top 5% by at least one other tool. Further, we observed that the higher the AUC, the more cases were present in the top 5% of scores (Additional file 2: Table S10). For the bottom 5% scores, the opposite is true; the higher the AUC, the fewer cases were present (Additional file 2: Table S10).

### Validation of PRS in the in-house IBD dataset

**Fig. 6:**
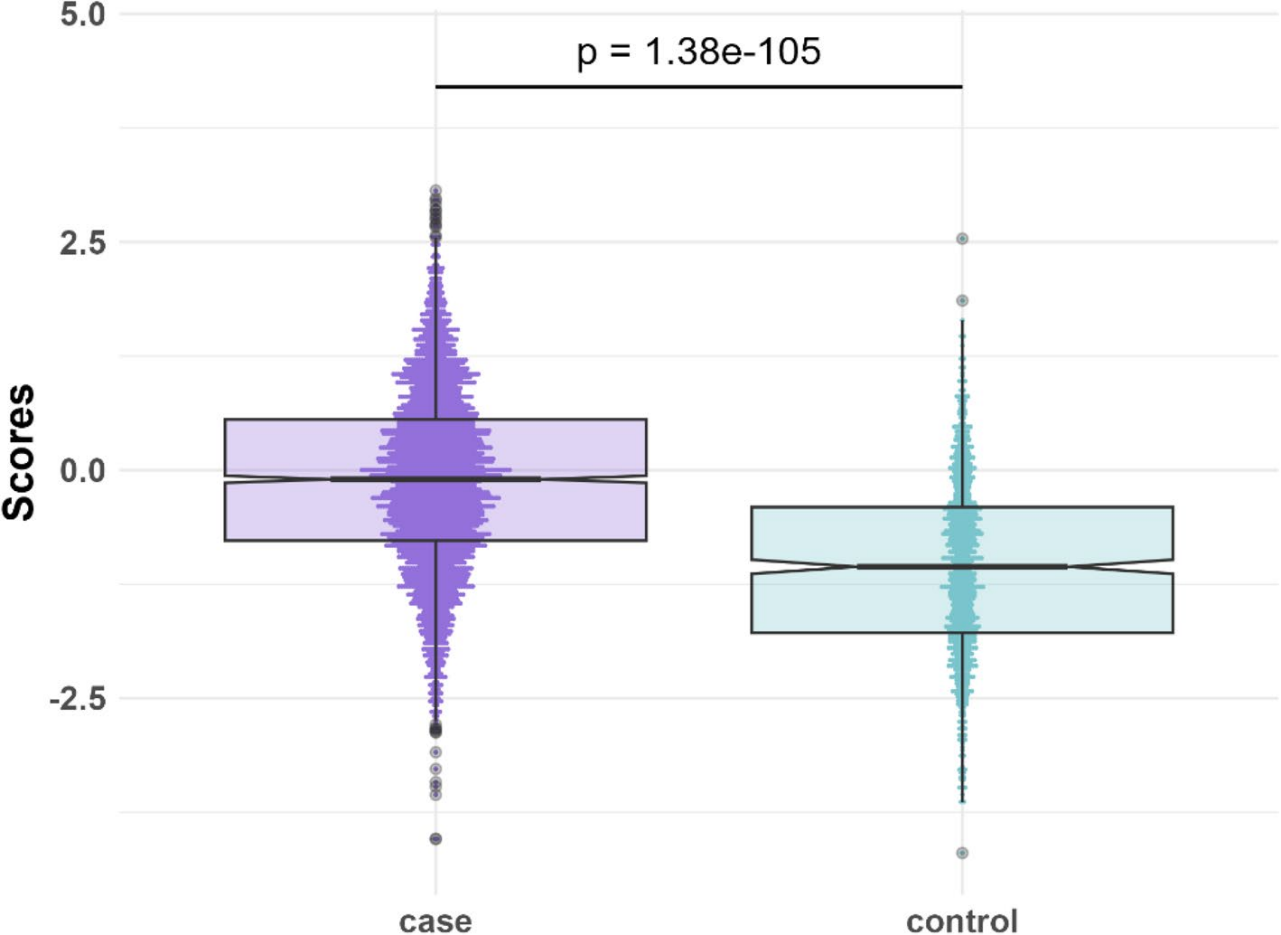
Boxplot showing the PRS of the IBD cases (purple) and controls (blue)

After determining the optimal parameter settings for each tool using the UK Biobank data (test dataset), we applied the optimal PRS to our in-house IBD dataset (validation dataset). The PRS Generated by lassosum, identified as the best-performing tool in the UK Biobank data, explained 20.3% of the variance in IBD in our in-house dataset, where each standard deviation increase in PRS is associated with a 2.6-fold increase in odds of having IBD (*OR* = 2.60 (*CI*: 2.38–2.85), *P*-value = 1.15e-94). Further, in the validation dataset, the mean PRS for IBD cases (−0.11) was significantly higher than for controls (−1.06, *P*-value = 1.38e-105) (Fig. 6).

The obtained *R*^2^ value in the validation dataset is notably higher than the *R*^2^ observed in the UK Biobank dataset, but this does not necessarily indicate better predictive performance, as the difference may be driven by the difference in case-to-control ratios between the two datasets. To further contextualize PRS performance in both cohorts, we estimated SNP heritability using LD score regression for both the UK Biobank (*h2* = 0.1102, *SD* = 0.0322) and our in-house IBD dataset (*h2* = 0.0295, *SD* = 0.1283). These estimates confirm that IBD has measurable heritability in both cohorts, which justifies evaluating PRS performance in each dataset.

Based on the confusion matrix, derived from the observed case prevalence in the validation dataset (Table 3) and ROC analysis, the IBD PRS achieved an overall accuracy of 0.6463, with a sensitivity of 0.6187, a specificity of 0.7516, and an AUC of 0.7507 (*CI*: 0.7321–0.7694) for distinguishing IBD cases from controls. While the PRS demonstrated a high positive predictive value (0.9048), its negative predictive value was considerably lower (0.3405). Incorporating covariates such as age and sex in the model resulted in a slight decrease in the accuracy (0.6225), sensitivity (0.5976), and specificity (0.7491). However, the positive predictive value increased to 0.9239, whereas the negative predictive value dropped further to 0.2676.

**Table 3:** Confusion matrices

**Only PRS**	**True case**	**True control**	**Metrics**	
**Predicted case**	1882	198	Sensitivity	0.6187
**Predicted control**	1160	599	Specificity	0.7516
			Accuracy	0.6463
			AUC (95% CI)	0.7507 (0.7321–0.7694)
			Positive predictive value	0.9048
			Negative predictive value	0.3405
**PRS + age + sex**	**True case**	**True control**	**Metrics**	
**Predicted case**	1772	146	Sensitivity	0.5976
**Predicted control**	1193	436	Specificity	0.7491
			Accuracy	0.6225
			AUC (95% CI)	0.7341 (0.7125–0.7556)
			Positive predictive value	0.9239
			Negative predictive value	0.2676

Top, logistic regression model with only PRS as independent variable. Bottom, logistic regression model with PRS and age and sex as independent variables

## Discussion

PRS calculation methods vary widely, with multiple tools available that use different approaches to handle LD and select SNPs. We developed a streamlined and flexible pipeline, STREAM-PRS, which integrates five popular tools, standardizes the scores, and automatically selects the best PRS for a given phenotype. Using IBD as a case study, we showed how STREAM-PRS reliably identified optimal parameter settings and achieved robust performance, particularly with lassosum, which explained 20.3% of the variance in IBD in our in-house dataset. Moreover, this PRS has a high positive predictive value (0.9048) but limited negative predictive value (0.3405). In terms of predicting IBD status, the PRS had an AUC of 0.75.

However, tool-specific challenges, such as genome build alignment (e.g., PRS-CS requiring build 37 or rsIDs) and missing GWAS summary statistics columns (e.g., “N” (i.e., sample size for the corresponding SNP) for lassosum, lassosum2, and LDpred2), were addressed but not automated in the pipeline, as these often vary between GWAS summary statistics. This highlights the need for harmonization when sharing GWAS summary statistics, a gap that remains despite resources like the GWAS catalog [33].

Another important consideration is the selection of SNPs going into the score. We observed that the variance explained by PRS was influenced by whether the SNPs overlapped with HapMap3 (Table 2). Using HapMap3 SNPs increased the overlap between training and validation datasets, thereby improving both PRS performance and portability. This is particularly relevant in clinical settings, where variations in genotyping methods can lead to differences in SNP availability across datasets. By restricting analyses to HapMap3 SNPs, we increase the chance that most SNPs are present across datasets, enhancing consistency and comparability of PRS results across datasets. However, this approach may exclude important disease-associated SNPs not present in HapMap3, such as those in the *NOD2* region for IBD (rs2066844, rs2066845, and rs2066847). This limitation was especially noticeable when fewer SNPs were included in the scores, where performance was better without restricting to HapMap3, suggesting that excluding high-impact SNPs can disproportionally affect PRS accuracy in certain contexts. Further investigation is needed to balance SNP inclusion for performance and portability.

Population stratification significantly impacts PRS results, even within relatively homogeneous populations. In our study, applying PC correction mitigated these effects, as shown in Fig. 2. Standardization, where scores were adjusted to a mean of zero and a standard deviation of one, further ensured comparability across tools and datasets. For our European-focused dataset, we used the 1 KG-NFE population for standardization. In theory, this approach is adaptable to any population, provided the training dataset reflects the genetic structure of the test dataset. This also illustrates the broader issue of transferability across ancestries in the PRS field. While STREAM-PRS can potentially be applied to different genetic ancestries, it requires matching training, test, and GWAS summary statistics. Hence, the pipeline should be run separately for each population, using the appropriate input files. The tools included in the pipeline have previously been evaluated in diverse ancestries, and studies have shown that they perform well when ancestry-specific GWAS data are available, although their performance may be reduced compared to that observed in European populations [34–37]. This indicates that STREAM-PRS likely will perform similarly under ancestry-matched conditions; however, future research is needed to prove this. Recent tools, such as the PGS calculator from the PGS catalog, address these challenges by automatically adjusting PRS according to an individual’s genetic ancestry [15]. For admixed individuals, we recommend tools specifically designed for such populations and that, for instance, consider local ancestry [38]. These specialized tools can more accurately capture genetic risk in such diverse populations, where standard approaches often fall short.

In our IBD use-case study, we showed that limiting analysis to HapMap3 SNPs improved portability but excluded some influential SNPs. The impact of excluding such SNPs remains unclear, particularly when correlated variants are retained. Replacing less significant HapMap3 SNPs with key disease-associated variants from the same region could potentially enhance performance and warrants further investigation.

When using biobank-scale datasets, case/control imbalances often arise, which can cause the Nagelkerke *R*^²^ values in logistic regression to be low. This occurs because the presence of many controls leads to a higher variability among controls than among cases. To address case-to-control imbalances, we performed downsampling analyses, adjusting the case-to-control ratio from 1:100 to 1:2. This increased *R*^²^ values but did not affect the identification of optimal parameter settings. Thus, the case-to-control ratio primarily influences absolute *R*^²^ values rather than tool or parameter selection. Furthermore, we estimated the heritability in both the UK Biobank and our in-house dataset. We observed that the heritability estimate in the in-house dataset was associated with a large SD, likely reflecting the smaller sample size, which limits the reliability of this estimate. Therefore, the observed differences in *R*^²^ between cohorts cannot be fully explained by the heritability estimates alone and may also be influenced by other factors such as sample size, case-control ratio, or cohort-specific characteristics. These findings nonetheless confirm that IBD has measurable heritability in both datasets, justifying and evaluating PRS performance in each. In addition, we calculated the Cox-Snell *R*^²^ as an alternative pseudo-*R*^*2*^ value and reached the same conclusion as with Nagelkerke *R*^²^. However, we note that differences in case-to-control ratios between datasets (e.g., UK Biobank vs. our in-house cohort) may still limit the comparability of absolute performance metrics across cohorts, particularly for prevalence-sensitive measures like Nagelkerke’s *R*^²^. This limitation should be considered when interpreting cross-cohort results.

Another challenge lies in the variability of PRS results across tools. Correlations between scores from different tools were Generally low, and there was limited overlap in individuals in the top or bottom 5% of the PRS distribution (Fig. 5). However, lassosum and PRS-CS showed higher correlations and overlaps, suggesting they may provide more consistent scores for IBD prediction. This variability highlights the importance of tool selection and parameter optimization in PRS analyses, as well as the need for caution when interpreting PRS results in clinical contexts. This caution was also highlighted in another study where they showed that uncertainty in PRS can have a large impact on risk stratification, which is considered a major application of PRS [39].

Identifying individuals at risk of disease using PRS must be approached with caution, as it could potentially cause significant stress and anxiety. In our IBD use case, the best-performing PRS demonstrated moderate sensitivity and specificity but high positive predictive value (Table 3). The negative predictive value, on the other hand, was low, suggesting that while the PRS effectively identifies individuals at higher risk for IBD, it is less reliable in excluding those at lower risk. This highlights the need for cautious interpretation of PRS in clinical settings, particularly given the differing case-to-control ratios across datasets. The addition of covariates to the model, here age and sex, did not improve the accuracy measures (Table 3). For IBD specifically, other covariates may provide greater predictive value, such as environmental risk factors (e.g., smoking behavior [40]) or inflammatory biomarkers like fecal calprotectin [41]. However, further research is needed to investigate whether adding these covariates alongside PRS could enhance prediction accuracy.

### Limitations and advantages of the pipeline

STREAM-PRS has several limitations. First, it cannot calculate PRS for multiple phenotypes simultaneously due to long runtimes, particularly for tools like PRS-CS. Second, we opted not to include the validation steps within the pipeline itself. This is because many researchers may not have access to validation datasets. Finally, while the pipeline is suitable for non-admixed populations, analyses involving admixed populations require specialized tools not yet integrated into the pipeline. For now, we refer to already existing tools for these purposes [38, 42, 43].

Despite these limitations, STREAM-PRS offers significant advantages, including flexibility in parameter selection, PC correction, and standardization. It generates multiple output files, allowing users to conduct additional analyses or create more plots, and provides an accessible platform for researchers new to PRS studies. By supporting multiple tools, it enables users to compare results and identify the best-performing PRS for their phenotype of interest.

## Conclusions

In summary, we present STREAM-PRS that integrates five widely used tools for PRS calculation, performs PC correction and standardization, and identifies the best PRS for a given phenotype. Our IBD use case demonstrated robust performance, highlighting the pipeline’s utility in optimizing PRS parameters across training, test, and validation datasets. We also highlighted the value of using HapMap3 variants to increase overlap between datasets, thereby enhancing PRS portability. Beyond its immediate applications, STREAM-PRS serves as a flexible and comprehensive framework that can be adapted to diverse phenotypes, populations, and research objectives, paving the way for broader adoption of PRS in personalized medicine and translational research.

## Supplementary Information

Additional file 1: Fig. S1. Overview of the STREAM-PRS pipeline, illustrating the workflow for the two use-cases. Fig. S2. Variance in IBD explained by the lassosum PRS for different control subsets from UK biobank. Fig. S3. Variance in IBD explained by the PRSice-2 PRS for different control subsets from UK biobank. Fig. S4. Variance in IBD explained by the PRS-CS PRS for different control subsets from UK biobank. Fig. S5. Variance in IBD explained by the LDpred2 PRS for different control subsets from UK biobank. Fig. S6. Variance in IBD explained by the lassosum2 PRS for different control subsets from UK biobank. Fig. S7. Violin plot of best PRS per tool for UK Biobank data. Fig. S8 Violin plot of best PRS per tool for UK Biobank data showing only the cases

Additional file 2: Table S1. Timing of running STREAM-PRS for 404 samples in training dataset and 1,000 samples in test dataset. Table S2. Regression results of all parameters for all tools on UK Biobank data for BMI PRS. Table S3: Summary of the correlations between the different scores and the overlap of individuals in the top/bottom 5% scores for BMI PRS. Table S4. Regression results of all parameters for all tools on UK Biobank data. Table S5: Regression results of PRS with lassosum on UK Biobank data. The regression was performed for 10 different control subsets. Table S6: Regression results of PRS with PRSice-2 on UK Biobank data. The regression was performed for 10 different control subsets. Table S7: Regression results of PRS with PRS-CS on UK Biobank data. The regression was performed for 10 different control subsets. Table S8: Regression results of PRS with LDpred2 on UK Biobank data. The regression was performed for 10 different control subsets. Table S9: Regression results of PRS with lassosum2 on UK Biobank data. The regression was performed for 10 different control subsets. Table S10: Summary of amount of cases in top/bottom 5% PRS based on UK Biobank data

## Data Availability

The PRS pipeline is available on GitHub: Becelaere S, Abakkouy Y, Jans DS, David M, Vermeire S, Cleynen I. STREAM-PRS: A multi-tool pipeline for streamlining Polygenic Risk Score computation. GitHub. [https://github.com/SaraBecelaere/STREAM-PRS](https:/github.com/SaraBecelaere/STREAM-PRS) [18]. The tools and their dependencies are available in a conda environment, which can also be downloaded from GitHub. Some of the tools and necessary files need to be downloaded separately before the tools can run. We refer to our GitHub page with instructions on how to download all necessary files.The summary statistics from the IBD GWAS and the weights of the effect sizes to replicate the polygenic scores are available from the KU Leuven research data repository upon request (doi:10.48804/R5KXWA) [44] .

## Abbreviations

1 KG-NFE: Non-Finnish European subset from the 1000 Genomes Project
AD: Alzheimer’s disease
AUC: Area under the receiver operating characteristic curve
BMI: Body mass index
C + T: Clumping and thresholding
CD: Crohn’s disease
CI: Confidence interval
GWAS: Genome-wide association studies
h2: Heritability estimates
IBD: Inflammatory bowel disease
LD: Linkage disequilibrium
MAF: Minor allele frequency
OR: Odds ratio
p: Proportion of causal variants
PC: Principal component
PGS: Polygenic score
PRS: Polygenic risk scores
Pts: *P*-Value thresholds
QC: Quality control
*R*^2^: Variance explained
ROC: Receiver operating characteristic
SD: Standard deviation
SE: Standard error
SNPs: Single-nucleotide polymorphisms
STREAM-PRS: Streamlined Toolkit for Reliable Evaluation and Analysis of Multiple Polygenic Risk Scores
UC: Ulcerative colitis
