# Supplementary material for: STREAM-PRS: a multi-tool pipeline for streamlining polygenic risk score computation
Source: Genome Med. 2025 Oct 9;17:119. doi: 10.1186/s13073-025-01539-0 (PMC12512491; doi:10.1186/s13073-025-01539-0)
Supplement: Supplementary file 1 — Additional file 1: Fig. S1. Overview of the STREAM-PRS pipeline, illustrating the workflow for the two use-cases. Fig. S2. Variance in IBD explained by the lassosum PRS for different control subsets from UK biobank. Fig. S3. Variance in IBD explained by the PRSice-2 PRS for different control subsets from UK biobank. Fig. S4. Variance in IBD explained by the PRS-CS PRS for different control subsets from UK biobank. Fig. S5. Variance in IBD explained by the LDpred2 PRS for different control subsets from UK biobank. Fig. S6. Variance in IBD explained by the lassosum2 PRS for different control subsets from UK biobank. Fig. S7. Violin plot of best PRS per tool for UK Biobank data. Fig. S8 Violin plot of best PRS per tool for UK Biobank data showing only the cases [file 13073_2025_1539_MOESM1_ESM.docx]

Additional file 1


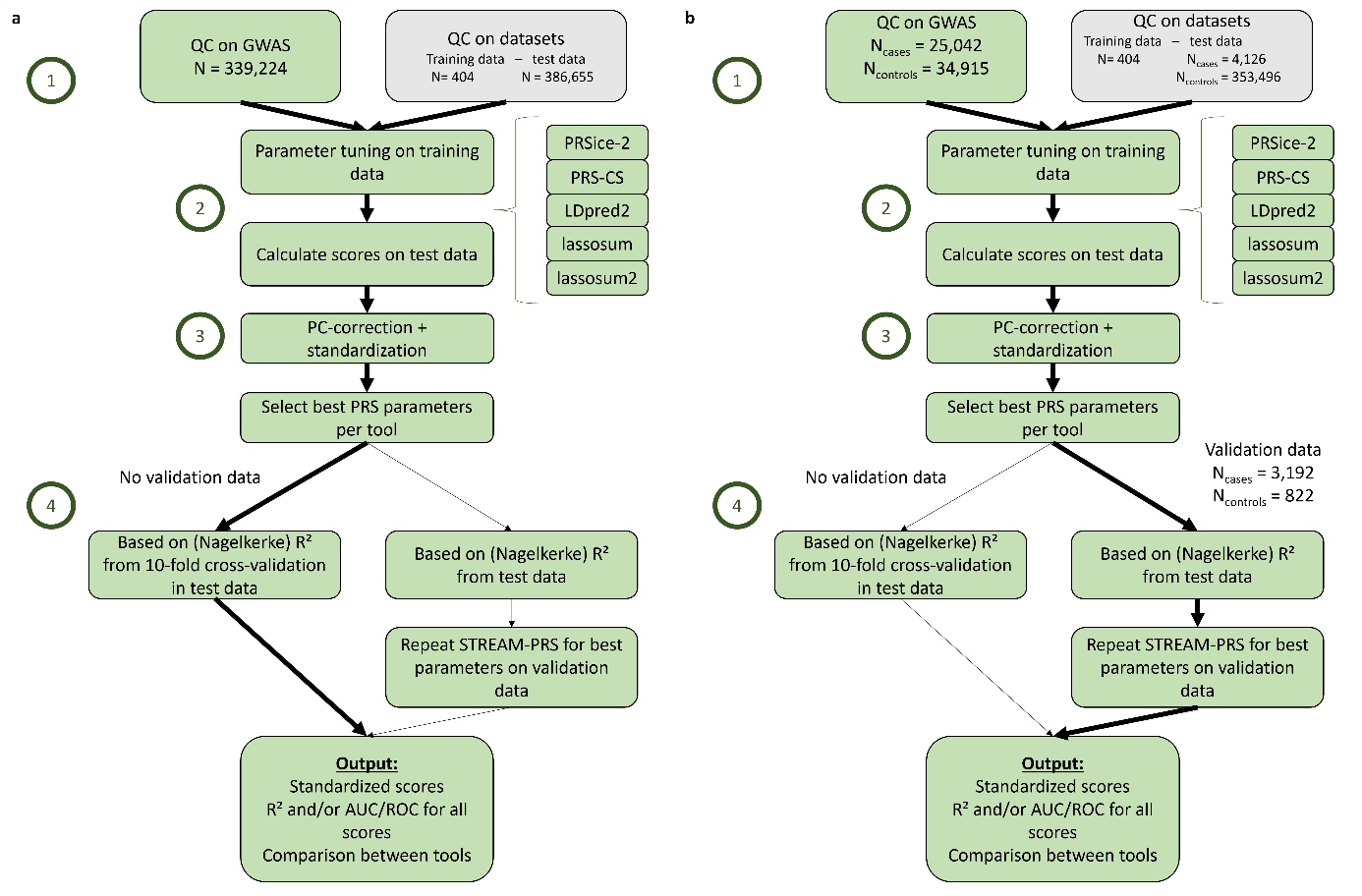


**Fig. S1. Overview of the STREAM-PRS pipeline, illustrating the workflow for the two use-cases.** The four main steps of the pipeline are indicated: (1) GWAS QC; (2) score calculation for the five included PRS tools (PRSice-2, PRS-CS, LDpred2, lassosum and lassosum2) by tuning the parameters in the training dataset and using these tuned parameters in the test dataset; (3) PC-correction and standardization of all scores; and (4) selection of best PRS. **a. BMI use-case.** For BMI, the GWAS by Locke et al. was used as input. The training dataset consisted of the non-Finnish European subset of the 1000 Genomes project and the test dataset was the UK Biobank. Since no independent validation dataset was available, we applied 10-fold cross-validation within the test dataset. **b. IBD use-case. For IBD,** as input GWAS, de Lange et al was used. The training dataset was again the 1KG-NFE, and the test dataset was the UK Biobank. Here, an in-house IBD dataset was available as a validation set. Therefore, we repeated STREAM-PRS on the validation dataset using only the best parameter settings determined from the test set, and the performance metrics were obtained from the validation data.


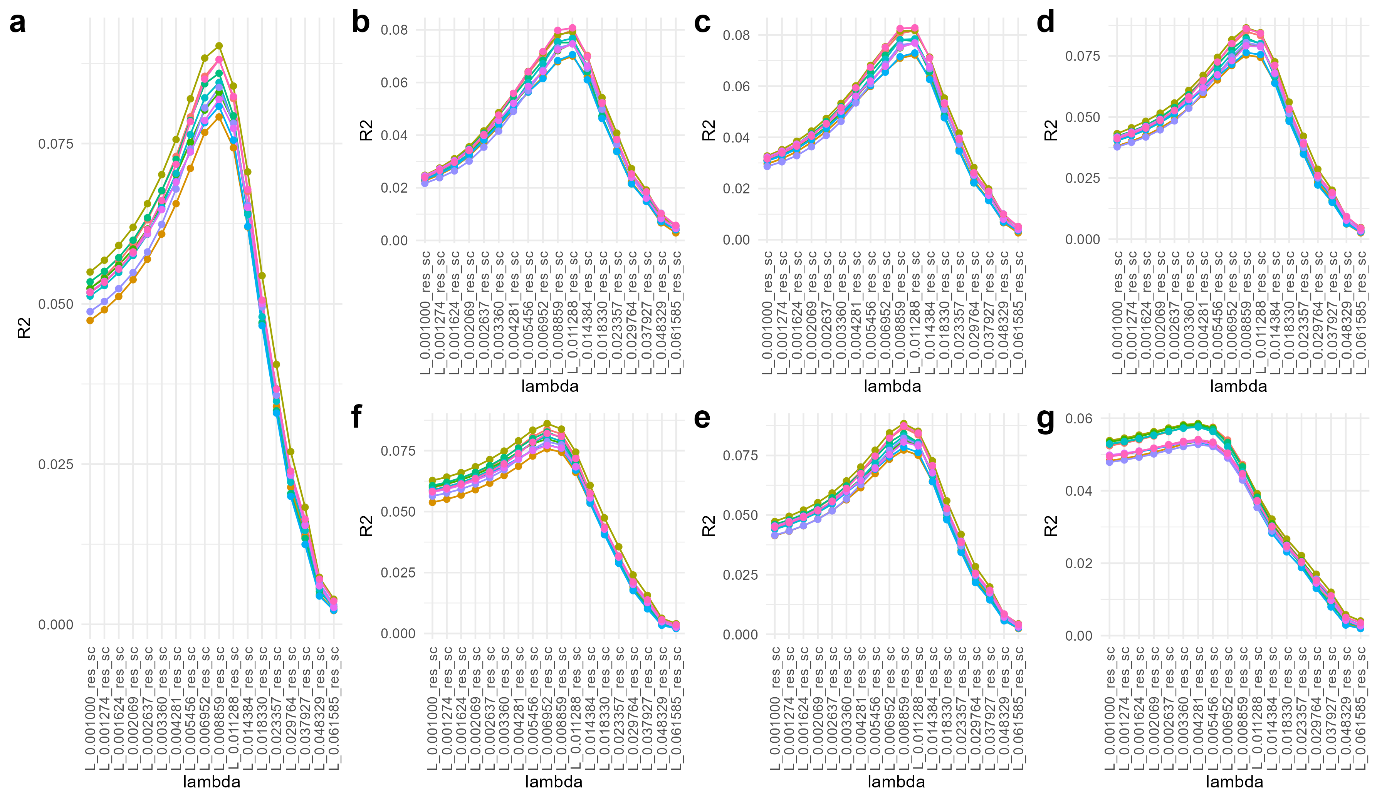


**Fig. S2. Variance in IBD explained by the lassosum PRS for different control subsets from UK biobank.** In the x-axis, different lambda values are indicated and on the y-axis is the corresponding R² value. **(a)** shrinkage 0.7 (best PRS results), **(b)** shrinkage 0.1, **(c)** shrinkage 0.2, **(d)** shrinkage 0.4, **(e)** shrinkage 0.5, **(f)** shrinkage 0.9, **(g)** shrinkage 1. The different colors represent different control subsets.


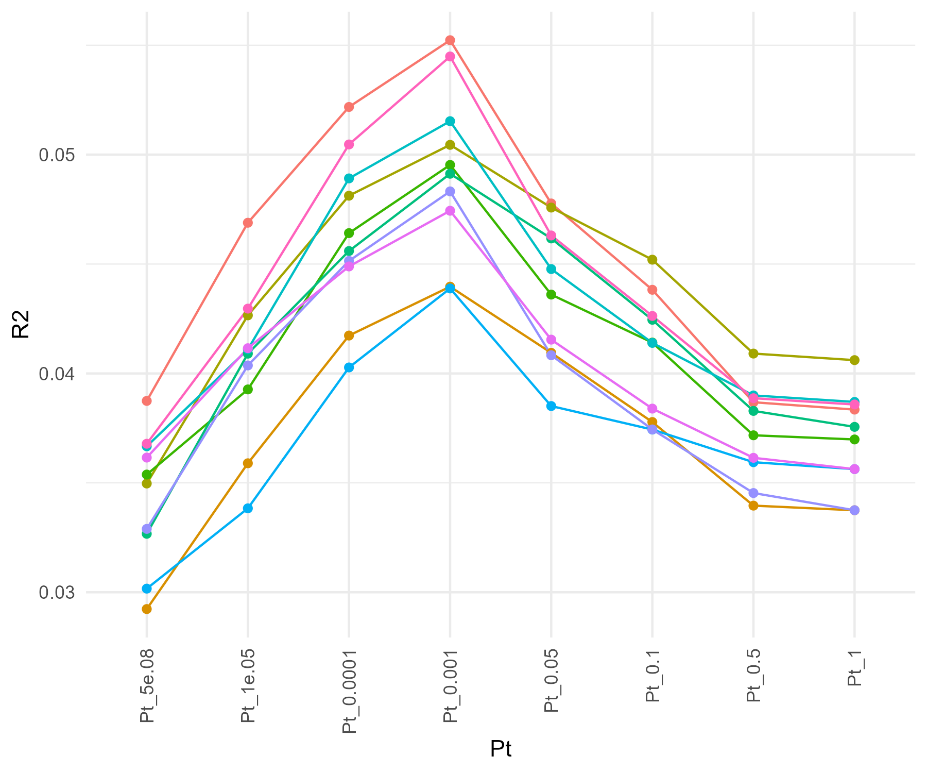


**Fig. S3. Variance in IBD explained by the PRSice-2 PRS for different control subsets from UK biobank.** In the x-axis, different P-value thresholds are indicated and on the y-axis is the corresponding R² value. The different colors represent different control subsets.


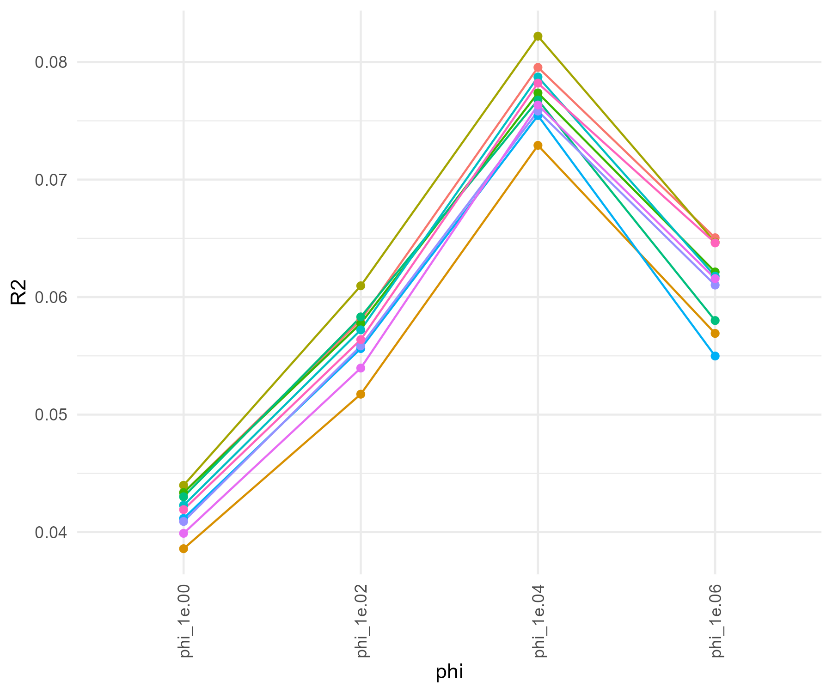


**Fig. S4. Variance in IBD explained by the PRS-CS PRS for different control subsets from UK biobank.** In the x-axis, different values for phi are indicated and on the y-axis is the corresponding R² value. The different colors represent different control subsets.


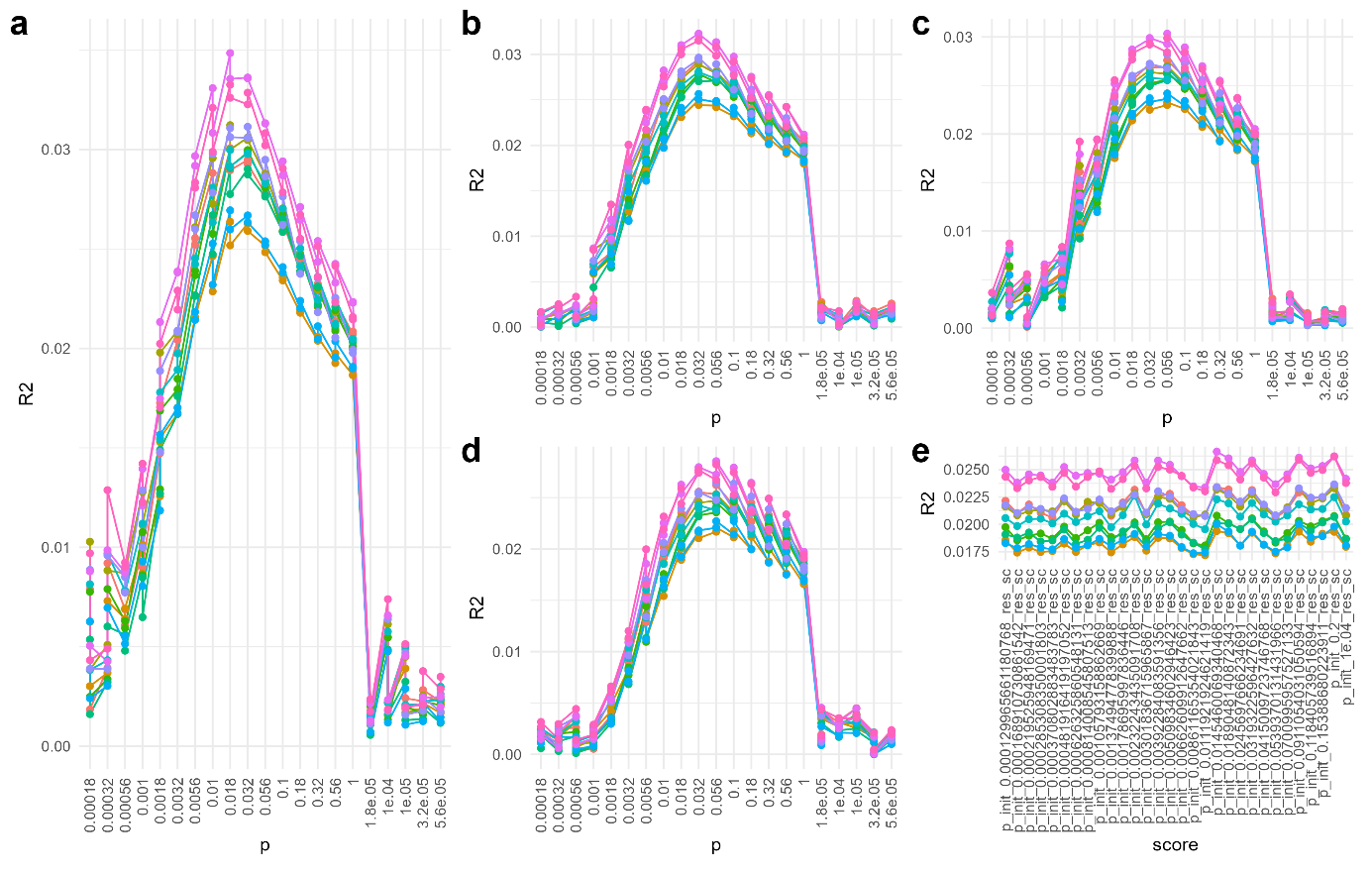


**Fig. S5. Variance in IBD explained by the LDpred2 PRS for different control subsets from UK biobank.** In the x-axis, different parameter settings are indicated and on the y-axis is the corresponding R² value. **(a-d)** grid model with h2 **(a)** 0.1873 (best PRS results), **(b)** 0.4370, **(c)** 0.6243, **(d)** 0.8741, **(e)** auto grid model with different initial values for p in x-axis. The infinitesimal and auto model are not shown, since they have no parameter options to change. The different colors represent different control subsets.


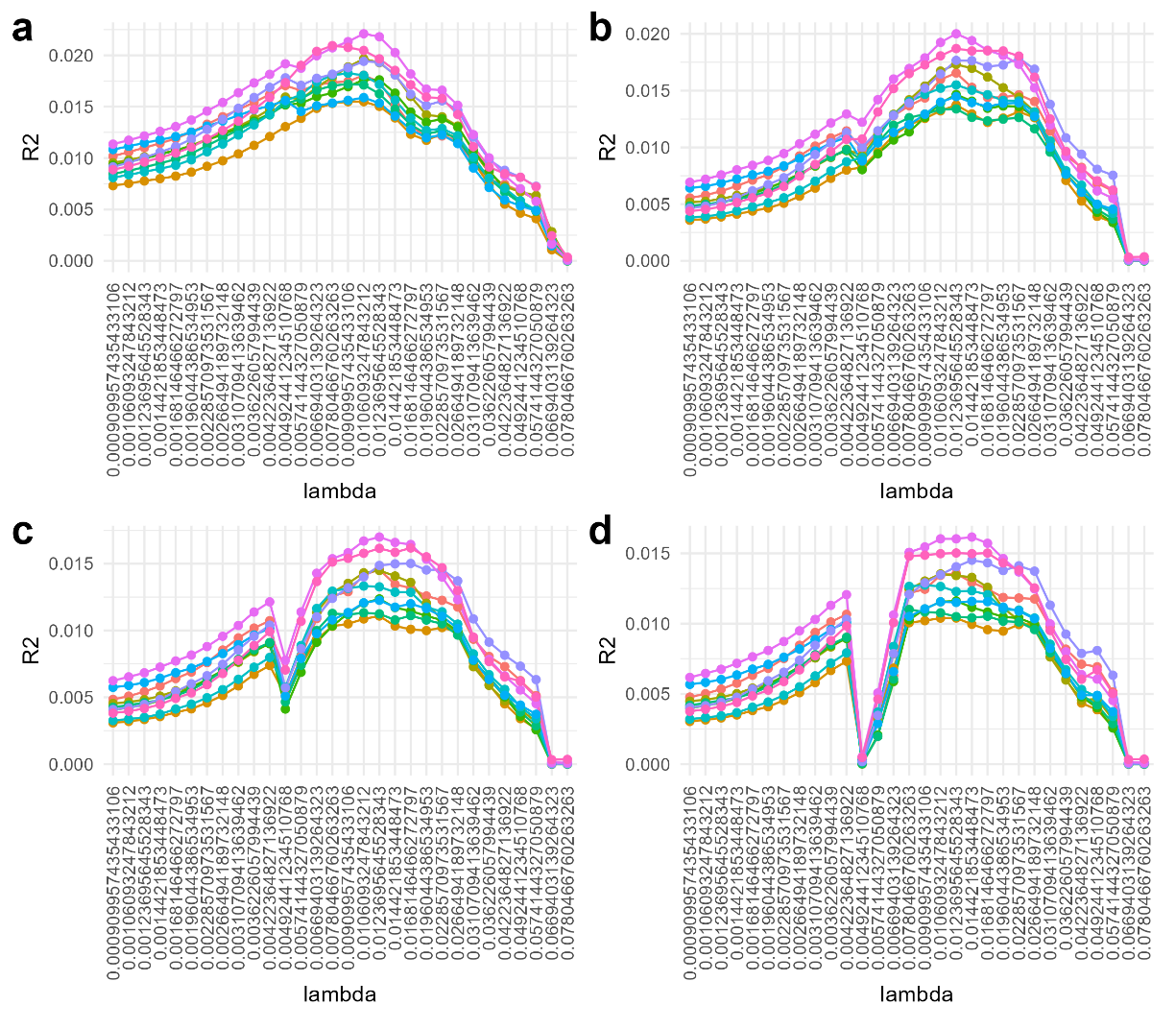


**Fig. S6. Variance in IBD explained by the lassosum2 PRS for different control subsets from UK biobank.** In the x-axis, different lambda values are indicated and on the y-axis is the corresponding R² value. **(a)** delta 1 (best PRS results), **(b)** delta 0.1, **(c)** delta 0.01, **(d)** delta 0.001. The different colors represent different control subsets


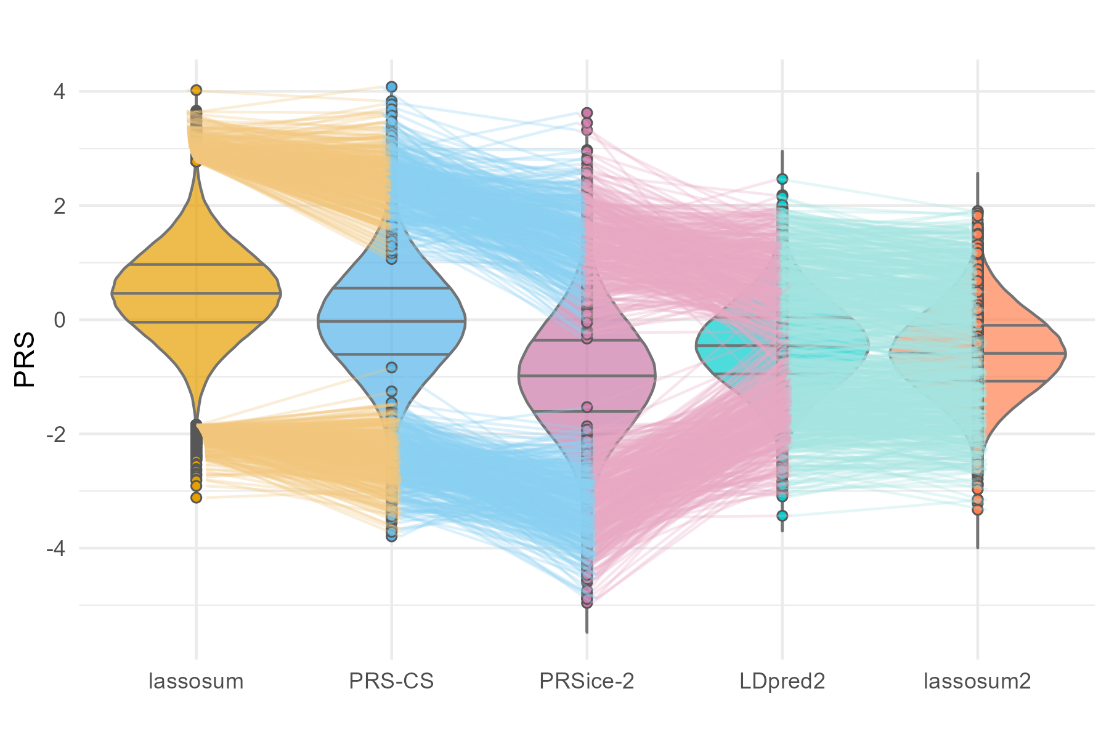


**Fig. S7. Violin plot of best PRS per tool for UK Biobank data.** The dots represent the top and bottom 0.1 percent individuals, ranked by PRS obtained from lassosum. Lines connect the same individuals.


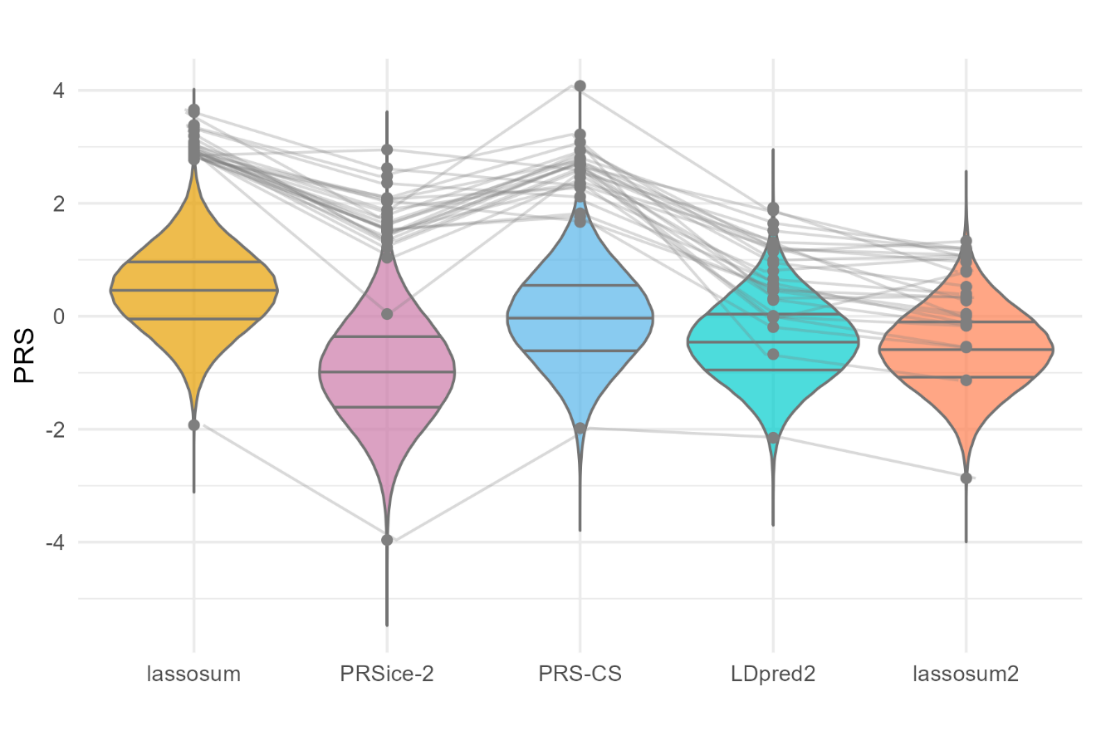


**Fig. S8 Violin plot of best PRS per tool for UK Biobank data showing only the cases.** The dots represent the cases that are present in the top and bottom 0.1 percent individuals, ranked by PRS obtained from lassosum. Lines connect the same individuals.
